# Supplementary material for: Extending the model of children’s conduct problems: A cross-sectional study of the interaction of maternal temperament and character, maternal parenting practices, and their child’s effortful control
Source: PLoS One. 2025 Aug 28;20(8):e0330897. doi: 10.1371/journal.pone.0330897 (PMC12393740; doi:10.1371/journal.pone.0330897)
Supplement: S1 Appendix — (DOCX) [file pone.0330897.s001.docx]

**S1 Appendix**

**Etch-A-Sketch – Cooperativeness Codes Description**

The first theoretical sub-scale that was translated into two codes was social acceptance vs. intolerance, which was designed as a bipolar scale. The two poles of the scale can be expressed by the same person in different situations at different times. Therefore, in practice, especially in an ecological setting, both types of behavioral manifestations can occur in different moments of the interaction between the mother and the child; hence, they can be coded as separate and not mutually exclusive scales.

Socially accepting individuals are accepting and respectful of other people’s ways, beliefs and perspectives, while intolerant individuals lack patience and acceptance towards those who hold different beliefs, opinions, or characteristics from their own. The concept of mothers' social acceptance was operationalized as a three-level code reflecting the degree to which the mother respects the child's opinion and accepts the child’s modes of action even if they differ from her own. For example, the mother listened to the child's opinion and took it into consideration. In cases when she thought that what the child suggested was not the correct way to approach the problem, she mediated this to the child in an appropriate and respectful manner. Moreover, she did not interrupt the child when they raised a claim and listened to it fully. In addition, she asked the child for their opinion. The coders rated 1 = *No expressions of social acceptance*, 2 = *Few expressions of social acceptance (one or two expressions)*, and 3 = *Many expressions of social acceptance (three expressions and above)*. The mothers' intolerance was operationalized as another three-level code reflecting the degree to which the mother imposed her way of action and ignored the child's suggestions. For example, the mother tried to dictate the rhythm of the task while ignoring the child's pace and rhythm; when the child suggested a way of action, the mother disrespected it, did not consider it or listen to it, and forced another way of action. The coders rated 1 = *No expressions of intolerance*, 2 = *Few expressions of intolerance (one or two expressions)*, and 3 = *Many expressions of intolerance (three expressions and above)*.

The second theoretical sub-scale that was translated into two codes was empathy vs. social disinterest. Similarly to the previous sub-scale, both poles of the scale were operationalized into two separated codes, as in ecological settings behavioral manifestations of both can occur in the same individual. Empathy was defined as a feeling of unity or identification with others, while social disinterest was defined as lack of interest, concern or an insensitive attitude in social interactions. The mothers' empathy was operationalized as a three-level code reflecting the degree to which the mother tried to understand and identify with the feelings and emotions of the child; for example, the degree to which the mother matched her responses to the child's emotional positive or negative state, or used empathic statements like "I can see it is difficult for you", "I see you are having fun!", etc. The coders rated 1 = *No expressions of empathy*, 2 = *Few expressions of empathy (one or two expressions)*, and 3 = *Many expressions of empathy (three expressions and above)*. The mothers' social disinterest was operationalized as a three-level code that reflected the degree to which the mother did not try to understand the feelings and emotions of the child or reacted in an insensitive manner; for example, the mother minimized or dismissed the feelings of the child (e.g., "Why are you so frustrated? It's only a game", "Don't get so angry", "It's not funny", etc.). The coders rated 1 = *No expressions of insensitivity*, 2 = *Few expressions of insensitivity (one or two expressions)*, and 3 = *Many expressions of insensitivity (three expressions and above)*.
